# The Pacific harbor seal gut microbiota in Mexico: Its relationship with diet and functional inferences

**DOI:** 10.1371/journal.pone.0221770

**Published:** 2019-08-29

**Authors:** Arlette Pacheco-Sandoval, Yolanda Schramm, Gisela Heckel, Elizabeth Brassea-Pérez, Marcel Martínez-Porchas, Asunción Lago-Lestón

**Affiliations:** 1 Centro de Investigación Científica y de Educación Superior de Ensenada, Ensenada, Baja California, Mexico; 2 Universidad Autónoma de Baja California, Ensenada, Baja California, Mexico; 3 Centro de Investigación en Alimentación y Desarrollo, Hermosillo, Sonora, Mexico; University of Illinois, UNITED STATES

## Abstract

Diet is a primary driver of the composition of gut microbiota and is considered one of the main routes of microbial colonization. Prey identification is fundamental for correlating the diet with the presence of particular microbial groups. The present study examined how diet influenced the composition and function of the gut microbiota of the Pacific harbor seal (*Phoca vitulina richardii*) in order to better understand the role of prey consumption in shaping its microbiota. This species is a good indicator of the quality of the local environment due to both its foraging and haul-out site fidelity. DNA was extracted from 20 fecal samples collected from five harbor seal colonies located in Baja California, Mexico. The V4 region of 16S rRNA gene was amplified and sequenced using the Illumina technology. Results showed that the gut microbiota of the harbor seals was dominated by the phyla Firmicutes (37%), Bacteroidetes (26%) and Fusobacteria (26%) and revealed significant differences in its composition among the colonies. Funtional analysis using the PICRUSt software suggests a high number of pathways involved in the basal metabolism, such as those for carbohydrates (22%) and amino acids (20%), and those related to the degradation of persistent environmental pollutants. In addition, a DNA metabarcoding analysis of the same samples, via the amplification and sequencing of the mtRNA 16S and rRNA 18S genes, was used to identify the prey consumed by harbor seals revealing the consumption of prey with mainly demersal habits. Functional redundancy in the seal gut microbiota was observed, irrespective of diet or location. Our results indicate that the frequency of occurrence of specific prey in the harbor seal diet plays an important role in shaping the composition of the gut microbiota of harbor seals by influencing the relative abundance of specific groups of gut microorganisms. A significant relationship was found among diet, gut microbiota composition and OTUs assigned to a particular metabolic pathway.

## Introduction

Harbor seals (*Phoca vitulina*) are the pinnipeds with the broadest distribution in the world, including the North Pacific and Atlantic oceans [1]. The southern limit of the distribution of the northern Pacific harbor seal (*P*. *v*. *richardii*) is located in Mexico on nine islands from west coast of Baja California: Coronado; Todos Santos; San Martin; San Jeronimo; San Benito; Cedros; Natividad; Asuncion; and, San Roque. This limit also comprises some areas along the Baja California coastline itself [2]. All of the islands are relatively close to the continental coast (2–50 km), making seals vulnerable to exposure to different disturbances and pollutants generated by human activity.

Harbor seals are considered opportunistic predators with a high trophic level that feed on benthic or pelagic or both species predominantly fish and cephalopods [3]. Mexican harbor seals feed at low depths, between 5–50 m [4,5], mainly on benthic or demersal prey [6–9]. Of the pinnipeds found in Mexico, the harbor seal has enabled the most amount of information to be obtained on the quality of their local environment, due to their fidelity to haul-out sites, where they remain all year [2]. Based on satellite transmitter data, it is now known that harbor seals forage near their colonies (at distance of between 25 km [10] and 30 km [4]); therefore, differences in the type of prey consumed are expected [8,9], because most of the harbor seal colonies in Baja California are separated by greater distances. However, differences in the diet have also been found in colonies separated by only 16 km [7]. This diet variability may be reflected in the seals’ microbiota, which may also reveal the presence of harmful substances in the environment.

The harbor seal diet is rich in proteins and polyunsaturated fatty acids [11], which is expected to be reflected in its gut microbiota composition [12]. As has been long established, diet largely determines the composition of the gut microbiota community [13,14] and is considered the main route of microbial colonization [15]. However, there are well known biases in traditional diet analysis techniques, especially in the identification of prey remains, due to digestive erosion [16]. Recently developed DNA techniques, such as DNA metabarcoding, have identified prey that leave no hard remains (*e*.*g*. crustaceans), providing more precise information on dietary ranges [16].

Gut microbiota plays an essential role in the host´s ontogenetic development, physiology, health, fitness, and nutrient assimilation [17,18]. Despite its importance, little is known about the composition of microbiota and its functions in marine mammals, in part due to sample collection difficulties. Various studies have examined the gut microbiota in captive or wild mammals, such as dugongs [19], manatee [20], whales [21], and pinnipeds [12,22–26], revealing a higher microorganisms richness than that found in their terrestrial counterparts, which could be due to the acquisition of marine bacterial taxa [27]. To our knowledge, only one study has explored the gut microbiota of harbor seals, but in semi-captive individuals [12]. Given that microbial composition is strongly influenced by environmental factors, the existing research, undertaken on captive animals, offers little scope for examining the effects of the gut microbiota on the host metabolism in the individual’s natural habitats, where factors, such as food availability and climate, vary [28].

Research in non-human primates has shown that gut microbiota studies are useful as indicators of the host's health and could help to detect genetic differences between populations [29]. As the microbial composition of individuals from the same population has more similarities than that of different populations [29], we expected to see differences among the microbiota community of the harbor seal colonies featured here. The aim of this study was to characterize the gut microbiota community of Baja California harbor seals, via the sequencing of the V4 region of 16S ribosomal microbial DNA taken from fecal samples, in order to understand the role of the diet on shaping the gut microbiome. We evaluated the relationship between the gut microbiome and diet as a first approach to understanding how the prey consumed shapes the composition and the function of the gut microbial community in wild harbor seals. This information may also position the harbor seal as an environmental sentinel species for the Baja California coast and its islands.

## Materials and methods

### Ethics statements

All samples were collected without disturbing any animal or causing them any stress or harm. Permits were provided by the Mexican Ministry for Environment and Natural Resources (SEMARNAT, SGPA/DGVS/12269/13 and SGPA/DGVS/08370/14), the Ministry of the Interior (SEGOB, UG/211/0087/2014 and UG/211/01022/2014), and the El Vizcaino Biosphere Reserve (F00.DRPBCPN.000027 and F.00.1.DRPBCPN.00004/2014).

### Sample collection

Twenty fecal samples were collected from May to June 2014 at five harbor seal colonies located in Baja California, Mexico: Punta Banda Estuary (PBE) (n = 5); Todos Santos Island (TS) (n = 3); San Jeronimo Island (SJ) (n = 4); Natividad Island (N) (n = 4); and San Roque Island (SR) (n = 4) (S1 Fig). The coordinates of the specific beaches where samples were collected are available in the metadata of the sequencing project in the database repository. Samples were collected during the molting season, when the highest numbers of individuals haul out [30] tending to remain on shore for many hours [31]. Each sample was collected individually using a disposable sterilized spatula. The samples were obtained from the inner part of the feces in an effort to avoid cross-contamination from the surrounding environment. The fecal samples came from seals without distinction of sex and age categories, with only fresh samples placed in sterile tubes and then preserved in RNAlater^®^ (Sigma-Aldrich). Samples were transported to the laboratory and kept frozen at -80°C until further analysis.

### DNA extraction and sequencing

Genomic DNA was extracted from 20 individual fecal samples including (PBE = 5, TS = 3, SJ = 4, N = 4 and SR = 4) using *QIAamp^®^ DNA Stool Mini Kit* (QIAGEN). Of each sample, approximately 300–400 mg of fecal material was used. DNA quality was visualized by agarose gel electrophoresis and the concentration was determined using a Nanodrop^®^ Spectrophotometer (Thermo Fisher Scientific).

The V4 hypervariable region of 16S rRNA gene was amplified by means of a two-step Polymerase Chain Reaction (PCR) method. The initial PCR was performed with 10 ng template DNA in a 20-μl reaction using the region-specific primers: 16S- F (5’- GTGCCAGCMGCCGCGGTAA- 3’) and 16-R (5’–GGACTACHVGGGTWTCTAAT- 3’) [32]. The PCR program consisted of an initial denaturation at 95°C for 3 min, and 25 cycles at 95°C for 30 s, 55°C for 30 s, and 72°C for 30 s, with a final extension at 72°C for 5 min. The second PCR was performed using primers from a Nextera XT Index Kit (Illumina) comprising eight cycles (95°C x 30 s; 61°C x 30 s; 72°C x 5 min). The PCR reactions were carried out individually for each sample, and the products were visualized by gel electrophoresis and quantified with a Qubit dsDNA BR Assay kit (Thermo Scientific) on a Qubit 3.0 fluorometer (Thermo Fisher Scientific). The normalization of PCR products was carried out using the SequalPrep^TM^ Normalization Plate (96) kit (Applied Biosystems^™^) according to the manufacturer’s protocol to obtain each amplicon at a concentration of 1–2 ng/ μl before mixing.

The sample pool (1100 pM), which contained 10 μl of each sample, was denatured with 0.2N NaOH, diluted further to 5 PM, and combined with 5% (v/v) denatured 5 pM PhiX, prepared adhering to the Illumina guidelines. The sequencing was performed at the sequencing facilities of the Center for Scientific Research and Higher Education at Ensenada (CICESE) using the MiSeq sequencing platform (Illumina). The chemistry used was the MiSeq Reagent Kit v2, yielding 2x150 bp paired-end reads. Initial quality control measures included the removal of any sequence containing an unresolved nucleotide and short sequences (< 100 bp).

### Sequence analysis

The paired-end assembled sequencing reads were quality-filtered (quality score 28), with singletons (unique sequences occurring only once) removed to minimize the effect of sequencing errors. Chimeric sequences were identified and removed by means of the UCHIME algorithm[33]. Sequence data was analyzed using the QIIME 1 software package [34], and the sequences were clustered into operational taxonomic units (OTUs) with an open-reference OTU picking protocol at the 97% sequencing identity level using UCLUST [35] against the SILVA 123 database. A total of 7211 OTUs were found in the 753,221 reads obtained. For downstream analyses, the OTU table was rarified to an even depth of 19,879 sequences per sample to avoid the biases generated by differences in sequencing depth.

Raw sequence data generated in the present study are available in the Read Archive of the National Center for Biotechnology Information (SRA NCBI) under the accession numbers PRJNA515402 (microbiome analysis) and PRJNA518147 (prey identification).

### Statistical analysis

The differences on effect of colony location on the relative abundance of bacterial phyla across samples were determined using ANOVA or Kruskal-Wallis when the data were not normally distributed in R 3.5 package [36], followed by a Tukey HSD or Dunn for multiple comparisons tests. The bacterial diversity either in the samples (alpha-diversity: Phylogenetic diversity) or between them (beta-diversity: UniFrac [37]) was calculated using QIIME [34]. The diversity index was compared via the R software [36] using an analysis of variance (ANOVA) or a Kruskal-Wallis, followed by a Tukey HSD, Duncan or Dunn multiple comparisons tests. A p< 0.05 was considered as statistically significant. Phylogeny-based (UniFrac) weighted and unweighted distances were used for exploring the beta diversity patterns and visualized using non-metric multidimensional scaling (nMDS) with the vegan package [38] in R 3.5 [36]. Comparisons across colonies were conducted using the Adonis function in QIIME on distance matrices with 999 permutations. The identification of the core microbiota, defined as the OTUs present in all samples (n = 20) with a relative abundance ≥ 0.5%, was performed in QIIME 1 using the comand *compute_core_microbiome*.*py*. A Bray-Curtis SIMPER analysis was performed in PAST 3.0.1 at both phylum and family level in order to determine which taxa explained the dissimilarities of the gut microbial communities and the possible functional variations observed among the different harbor seal colonies.

### Diet analysis

The diet analysis previously conducted on the harbor seals colonies of Todos Santos, San Jeronimo, Natividad, and San Roque islands had been characterized via metabarcoding analysis [9]. The same fecal samples were used in this study to characterize the gut microbial composition and complete the diet characterization. Therefore, in order to compare microbiota and diet for the five colonies of interest, genomic DNA from the PBE samples was extracted following the same methodology described in [9]. Briefly, we used a two-step PCR approach for each pair of marker primers including the V9 region of the rRNA 18S gene, for eukaryotes in general [39], and the 16S mitochondrial region for chordates [40] and cephalopod species, in particular [41]. A blocking oligonucleotide matching harbor seal sequences was used in the first PCR to prevent the amplification of the seal DNA [40]. Normalization and sequencing were undertaken in the same way as described for the microbiome libraries.

The implementation of the FastQ joiner and FASTX-toolkit in the Galaxy online interface was used to join the forward and reverse reads and to collapse identical sequences, respectively [42]. The assignment of prey species was performed using BLAST [43] against the non-redundant Genbank database with an E-value cut-off of 10^−20^, a percentage identity of ≥ 96% and an alignment length of ≥ 100 bp. The species assignments obtained were inspected manually to remove those species not distributed across the study area, the contaminants identified, species that were not potential prey (terrestrial animals, microorganisms, etc), and harbor seal sequences. The databases used to determine the distribution and habits of the prey identified were FishBase (http://www.fishbase.org/search.php) and SealLifeBase (http://www.sealifebase.org/). We used the R package UpsetR [44] to visualize the number of prey individuals, both shared and unique among the harbor seal colonies.

### Functional prediction

Closed reference OTU picking was performed against the GreenGenes (version 13.5) reference database in QIIME. The Langille Lab´s bioinformatics software package, PICRUSt (phylogenetic investigation of communities by reconstruction of unobserved states) [45], was applied on the Galaxy server and was used to predict the metagenome composition of the community via the 16S rDNA sequencing data. Once the 16S copy number was normalized and the metagenome functions had been predicted, KEGG (Kyoto Encyclopedia of Genes and Genomes) pathways were used to identify the metagenomic contents. Statistical comparisons were performed in the R software [36] using an ANOVA test followed by a Tukey HSD for multiple comparison test.

### Relationship between diet and gut microbiota

To assess the relationship between diet and gut microbial function and composition, we applied Spearman correlation tests between the prey frequency of occurrence, as indicated by the relative abundance of bacteria (five most abundant phyla and the 10 most frequently detected families) and the relative abundance of metabolic pathways. Those prey families and species with a >20% frequency of occurrence were included while a p< 0.05 was considered statistically significant.

## Results and discussion

### Dataset—General description

The microbiome dataset used in the present study comprised 20 samples taken from five different harbor seal colonies—PBE (n = 5), TS (n = 3), SJ (n = 4), N (n = 4) and SR (n = 4)—and a total of 753221 obtained sequences. The number of sequences per sample varied from 19879 to 52601 with an average of 911 OTUs identified in each sample. The PBE diet dataset contained 348306 collapsed sequences, 38% of which corresponded to the 18S rRNA marker for eukaryotes, and 32% and 29% to the 16S mtDNA marker for chordates and cephalopods, respectively.

### Taxonomic composition of harbor seal gut microbial communities

**Fig 1:**
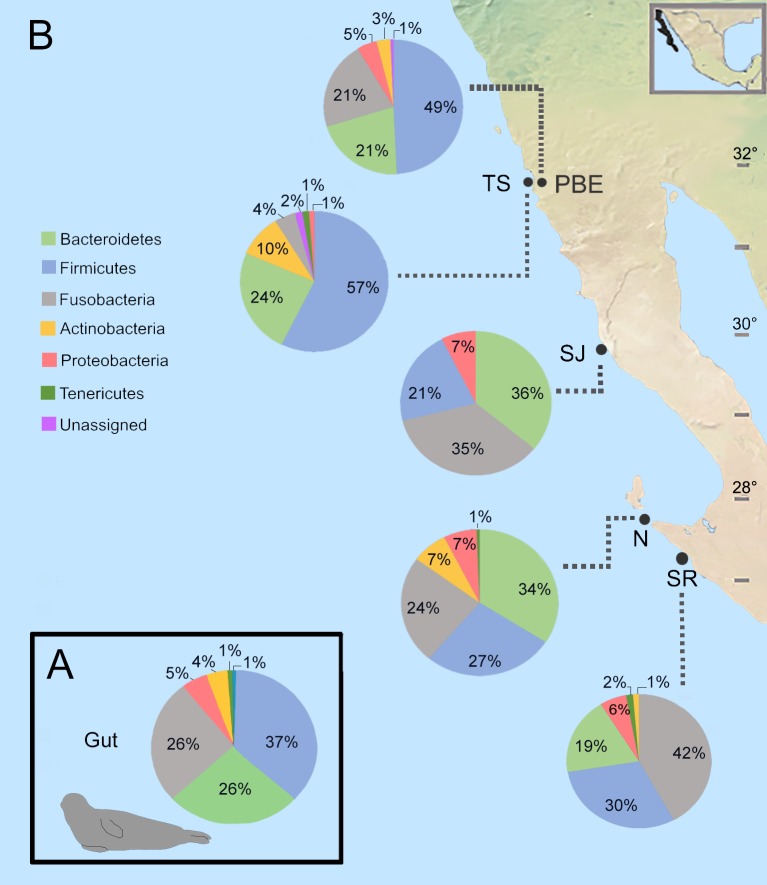
Bacterial phyla in harbor seal gut microbiota. (A) Overall composition of gut microbiota at the phyla level. (B) Average relative abundance of the most common bacterial phyla in the harbor seal colonies, only taxa with >1% relative abundance are shown. Harbor seal sampling locations on the western coast of Baja California are included in the figure as PBE = Punta Banda Estuary, TS = Todos Santos Island, SJ = San Jeronimo Island, N = Natividad Island, SR = San Roque Island.

Members of 26 phyla were detected in the gut microbiota of harbor seals with Firmicutes (37%), Bacteroidetes (26%), Fusobacteria (26%), Proteobacteria (5%), and Actinobacteria (4%) as the main phyla shaping the gut microbial diversity in the harbor seals (Fig 1A) (S1 Table). The dominance of these phyla in the harbor seal´s gut microbiota has been reported previously [12,25]. Like other marine mammals, the harbor seal presents a lower abundance of the phylum Firmicutes than that presented by terrestrial mammals [26]. A higher proportion of Firmicutes than Bacteroidetes has been related to both a greater efficiency in harvesting energy from food and occurrence of obesity in humans and mice [46]. The high Firmicutes to Bacteroidetes ratio observed in the gut of harbor seals could be due to the fact that these animals heavily rely on fat deposition for energy storage and thermoregulation [22,47]. Thus, it is possible that harbor seals rely on their gut microbes to facilitate the efficient fat storage needed for survival, via the increased availability of colonic energy, as observed in humans [48].

**Fig 2:**
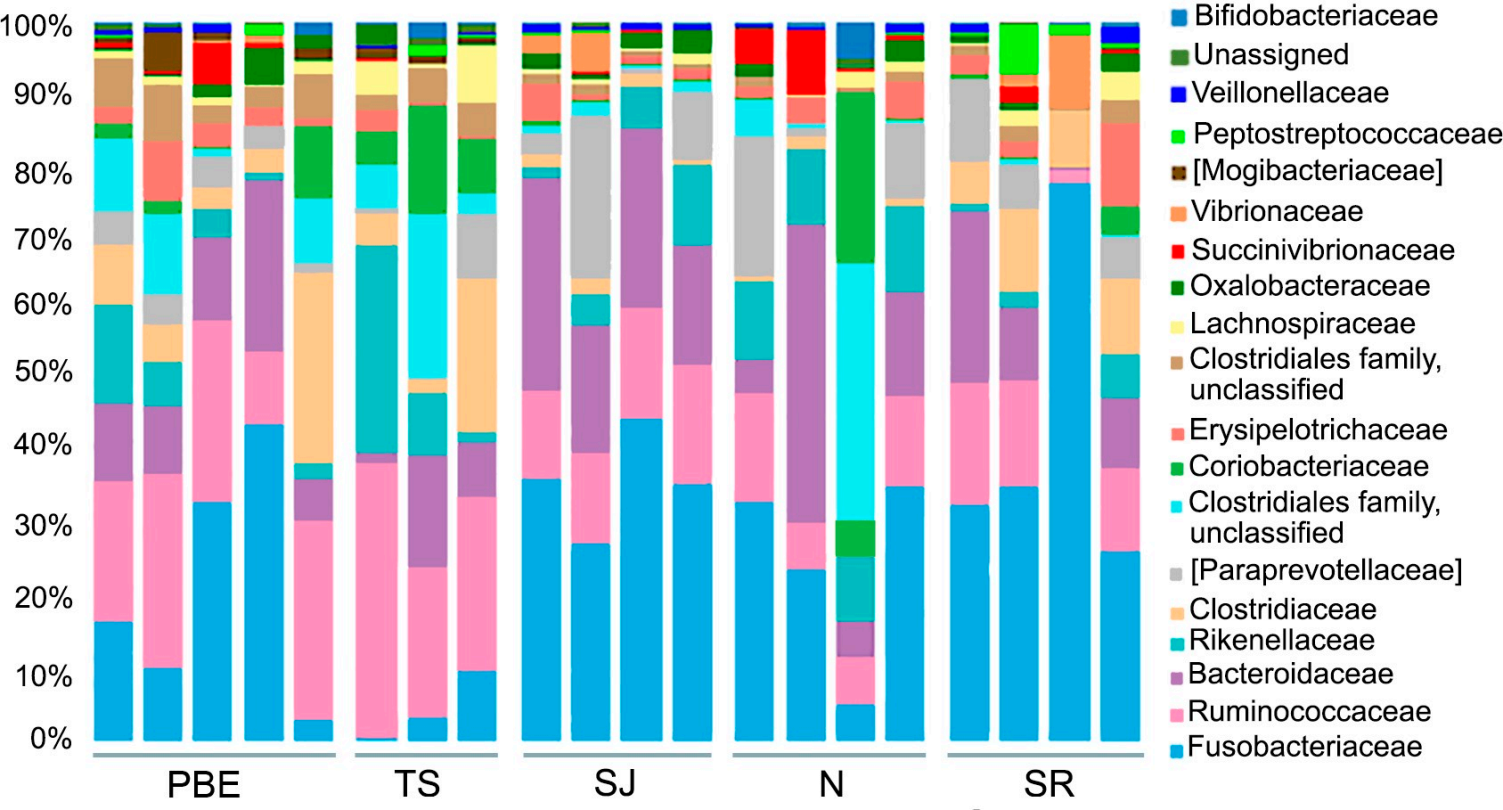
Composition of gut microbiota at family level. Only taxa with a >1% relative abundance are shown. PBE = Punta Banda Estuary, TS = Todos Santos Island, SJ = San Jeronimo Island, N = Natividad Island, SR = San Roque Island.

At the bacterial family level, 19 families were identified with a relative abundance of ≥ 1% and included the bacterial order Clostridiales, although it was not classified at the family level. Fusobacteriaceae was the most abundant family (27%), followed by Ruminococcaceae (16%), and Bacteroidaceae (14%) (Fig 2). Members of the family Ruminococcaceae have presented a strong positive correlation with polyunsaturated triglycerides of dietary origin [49], which are abundant in harbor seal prey, such as the anchovy and the sardine.

*Fusobacterium* (phylum Fusobacteria), a genus known for displaying proteolytic activity in the human gut [47], was found to be the predominant genus in the gut microbiota of harbor seals (25%). Marine mammals have a higher abundance of Fusobacteria than terrestrial mammals [26]. Members of this phylum, such as *Fusobacterium*, inhabit the oral cavity and gut of animals as well as being found in marine sediments [18]. Harbor seals may acquire these microorganisms from the microbiota of sediment as they mainly feed on benthic and demersal prey (e. g. flatfishes) [6,50]. The acquisition of these microbes could bring useful new genes from the global microbiome to the gut microbiome via horizontal gene transfer [18].

Within the phylum Bacteroidetes, the genera *Bacteroides* (14%), and other representatives of the Rikenellaceae (7%) and Paraprevotellaceae (6%) families were also found to be abundant. The genus *Bacteroides* has been related to carnivorous diets, characterized by high levels of proteins, amino acids and animal fats [51]. Encoding a large number of animal-derived carbohydrate-active enzymes, *Bacteroides* have a high capacity to hydrolyze dietary glycans, a type of polysaccharides derived from proteins [52]. Therefore, the high abundance of the genus *Bacteroides* found in harbor seals by the present research is consistent with their carnivorous habits. The enzymes contained in these types of bacteria may assist the harbor seal to obtain energy from their consumed prey [51].‬‬‬‬‬‬‬‬‬‬‬‬‬‬‬‬‬‬‬‬‬‬‬‬‬‬‬‬‬‬‬‬‬‬‬‬‬‬‬‬‬‬‬‬‬‬‬‬‬‬‬‬‬‬‬‬‬‬‬‬‬‬‬‬‬‬‬‬‬‬‬‬‬‬‬‬‬‬‬‬‬‬‬‬‬‬‬‬‬‬‬‬‬‬‬‬‬‬‬‬‬‬‬‬‬‬‬‬‬‬‬‬

The Firmicutes were represented by members of Ruminococcaceae (14%), Clostridiaceae (3%), Lachnospiraceae (1%), Mogibacteriaceae (1%), and Peptostreptococcaceae (1%) families, and by the genera *Clostridium* (3%), *Oscillospira* (1%), *Faecalibacterium* (1%) and *Phascolarctobacterium* (1%). The Ruminococaceae and Lachnospiraceae families play important roles in host immune functions [53] and are commonly found in the digestive tracts of many mammalian species, while abundant populations are absent in non-host associated environments [54–56].

The phylum Proteobacteria was mainly represented by the genera *Cupriavidus* (1%), and *Anaerobiospirillum* (1%), and by a member of the family Vibrionaceae (1%). To a lesser degree, we found members of this phylum not commonly considered as present in the digestive tract of mammalian animals, the genera *Photobacterium* (0.004%) and *Vibrio* (0.0005%), which are reported in higher abundance in animals with squid-based diets, such as pilot whales [57]. Luminescent bacteria *Photobacterium* and *Vibrio*, are common members of the squid microbiota and may be acquired by harbor seals via squid consumption [58]. *Atopobium* (3%) and *Slackia* (1%) were the main representatives of the phylum Actinobacteria.

The analysis of the microbial community conducted on all samples in the present research, suggests the presence of a core microbiota in harbor seals. Of the 1,041 OTUs detected, only 32 OTUs were present in all samples with twenty-one of these core OTUs belonging to the phylum Firmicutes, six to Bacteroidetes, four to Fusobacteria and one to Actinobacteria. These include representatives of the Ruminococcaceae (14), Fusobacteriaceae (4), Rikenellaceae (3), Clostridiaceae (3), Erysipelotrichaceae (2), Bacteroidaceae (2), Lachnospiraceae (1), [Paraprevotellaceae] (1), and Coriobacteriaceae (1) families, as well as a member of the order Clostridiales. The abovementioned families are abundant in other pinnipeds [22,24]. The 32 OTUs represent up to 62% of the microbial community observed, although their relative abundance varied between samples. The genera *Clostridium*, *Bacteroides*, *Fusobacterium*, and *Faecalibacterium*, have also been identified in the core microbiota of other phocid seals [10,36]. The genera *Clostridium*, *Bacteroides*, *Fusobacterium*, *Prevotella*, and *Faecalibacterium* have also been identified by another study as the core microbiota of harbor seals from the Baltic Sea [12], although the genera *Oscillospira*, *Eubacterium* and *Atopobium* were not found in the Atlantic harbor seal population (S2 Table). These microbiota members may be seal-specific and may play an important role in the host’s evolution, metabolism, and immunity [23] with their vertical transmission from mother to pup ensuring the maintenance of this core community [12].

### Comparision between colonies

As the gut microbial composition of individuals from the same population present more similarities than that of individuals from different populations [29], we expected to find differences in the microbiota of the different harbor seal colonies studied here. Our results showed variations among the harbor seal colonies in terms of the relative abundance of the dominant phyla (S2 Fig). Of the 26 phyla observed, five contributed >2% to the differences found among colonies, based on the results obtained from the SIMPER analysis (Table 1). Changes in relative abundance of Firmicutes (33.88%), Fusobacteria (31.95%), and Bacteroidetes (16.86%) members, mainly explain the diversity differences observed (Table 1). According to the SIMPER analysis, at a family level Fusobacteriaceae (25.93%) and Ruminococcaceae (13.19%) contributed most to the differences in gut microbiota found among the harbor seal colonies (Table 1). Members of the families Ruminococcaceae and Lachnospirace are common in the mammalian gut and are known to transform carbohydrates into short-chain fatty acids (SCFA) via microbial fermentation, with the end-product absorbed by the host as an energy source [59].

**Table 1:** SIMPER analysis results comparing the gut microbiota composition of the studied harbor seal colonies^a^. PBE = Punta Banda Estuary, TS = Todos Santos Island, SJ = San Jeronimo Island, N = Natividad Island, SR = San Roque Island.

Taxon		Mean abundance (%)				
	Contribution (%)^b^	PBE	TS	SJ	N	SR
**Phylum**						
Firmicutes	33.9	48.9	57.3	21.3	27.0	30.4
Fusobacteria	32.0	21	4.5	34.8	23.8	41.9
Bacteroidetes	16.9	20.9	24.2	35.5	33.9	18.7
Actinobacteria	8.9	3.2	9.8	0.4	7.4	1.3
Proteobacteria	5.2	4.6	1.2	7.4	6.8	6.0
**Family**						
Fusobacteriaceae	25.9	21.0	4.5	34.8	23.8	41.9
Ruminococcaceae	13.2	21.4	27.2	13.8	9.9	10.9
Bacteroidaceae	9.4	11.9	7.9	21.3	15.9	10.7
Clostridiales family, unclassified	8.3	6.2	10.0	1.3	10.2	0.3
Rikenellaceae	7.5	5.2	12.6	5.5	10.4	2.3
Clostridiaceae	6.5	9.1	9.1	1.6	2.0	8.6
Coriobacteriaceae	6.3	2.9	8.8	0.3	5.9	1.2
[Paraprevotellaceae]	4.2	3.5	3.4	8.5	7.6	5.7
Clostridiales family, unclassified	3.3	5.0	3.8	1.1	0.8	1.6
Succinivibrionaceae	2.3	1.3	0.1	0.2	3.6	0.6
Lachnospiraceae	2.3	1.2	4.3	0.6	1.1	1.6
Vibrionaceae	2.2	0.3	0.0	1.9	0.0	2.9
Erysipelotrichaceae	2.1	3.4	1.2	2.1	2.5	4.1

^a^ The Bray-Curtis average dissimilarity values was >1% for this taxa.

^b^ Contribution to the dissimilarity between harbor seals’ colonies.

The northern colonies (PBE and TS) presented the highest proportions of the phylum Firmicutes, while OTUs from the family Ruminococacceae (27% ± 21%) contributed to the increase observed (Table 1). The microbial profile obtained for TS presented the highest abundance of Firmicutes (57%) and Actinobacteria (10%) and the lowest proportions of Fusobacteria (4%) and Proteobacteria (1%) (Fig 1B).

On San Jeronimo and Natividad islands, the harbor seals’ microbial communities contained a higher abundance of the phylum Bacteroidetes (36% ± 34%), with Bacteroidaceae family contributing most to these proportion. The lowest abundance of Actinobacteria was observed on San Jeronimo Island (< 1%), while the southern colony, San Roque Island, presented the highest abundance of the phyla Fusobacteria (42%) and Tenericutes (2%) (Fig 1B), of which the Fusobacteriaceae (42%) and Mycoplasmataceae (1%) families were the most representatives, respectively. SIMPER analysis conducted at OTU level indicated that Fusobacterium members contributed the most to the gut microbiota dissimilarity found among the harbor seal colonies (S3 Table), with these OTUs being the most abundant in the fecal samples collected on San Roque Island.

**Fig 3:**
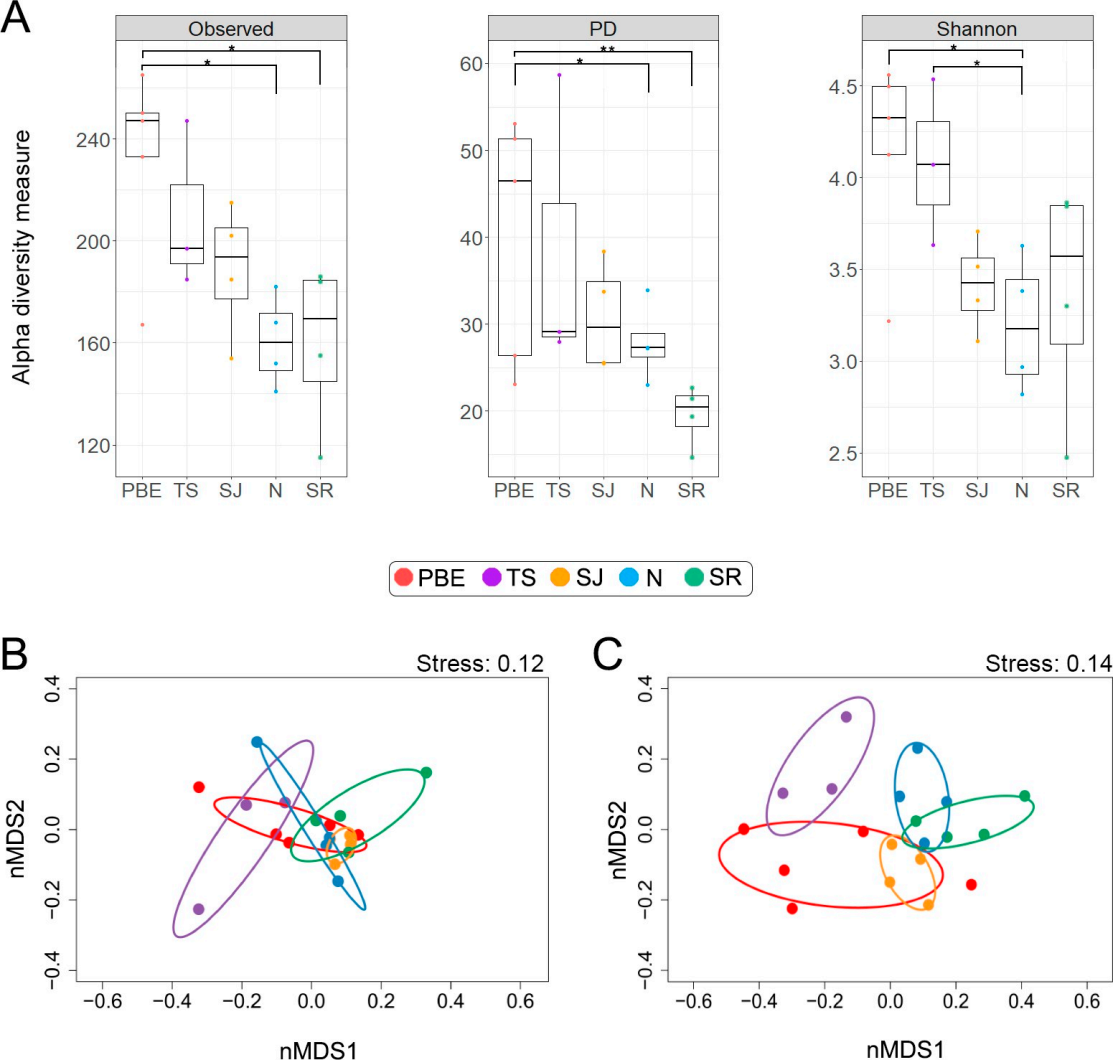
Comparison of the gut microbiome of the different harbor seal colonies. (A) Alpha diversity was measured by means of observed OTUs, phylogenetic diversity (PD) and Shannon index values. Non-metric multidimensional scaling analysis (nMDS) conducted for the weighted (B) and unweighted (C) UniFrac distance matrix. Ellipses correspond to a confidence area of 98% based on the standard error for the weighted and unweighted UniFrac metric. PBE = Punta Banda Estuary, TS = Todos Santos Island, SJ = San Jeronimo Island, N = Natividad Island, SR = San Roque Island. *p <0.05, **p<0.01.

We observed statistical differences in OTU richness among the harbor seal colonies (ANOVA = 0.014), wherein the harbor seals of Punta Banda Estuary showed a higher OTU richness than the seals inhabiting Natividad (Tukey HSD = 0.025) or San Roque (Tukey HSD = 0.023) islands (Fig 3A). The diversity of harbor seal gut microbiota varied among colonies, according to the mean phylogenetic diversity (PD) (Kruskal-Wallis: p = 0.004) and Shannon index (ANOVA: p = 0.03) (Fig 3A) values. Differences in the PD index values were observed between PBE-N (Dunn: p = 0.01) and PBE-SR (Dunn: p = 0.005), while Shannon index differences were found between PBE- N (Duncan: p = 0.02) and TS-N (Duncan: p = 0.03).

Higher values of the gut microbiota diversity and richness were observed in the harbor seals from PBE, which is the only coastal harbor seal colony studied in the present research and is the colony with the lowest number of individuals. Previous studies conducted in pinnipeds have shown that high-density colonies present significantly higher microbial diversity than low-density colonies due to the higher potential for microbial transmission [22,23]. However, our results do not support these findings, suggesting that other factors may play a major role in shaping the gut microbiota of the PBE harbor seals. The acquisition of environmental bacteria may be more prominent in the harbor seals from this colony due to their proximity to the land and their contact with streams and wastewater runoff, which could be sources of associated bacteria. Moreover, PBE is the colony most frequently visited by humans, which may increase bacterial transmission.

Comparisons of the members of the gut microbiota community studied here showed that colony location significantly influenced the overall structure (weighted UNIFRAC) (adonis: R^2^ = 0.28; p ≤0.001) and membership (unweighted UNIFRAC) (adonis: R^2^ = 0.33; p ≤0.013) of the fecal microbiota of the harbor seals (Fig 3B and 3C). Colony dynamics, behavior and foraging site could be factors in shaping the gut microbiota of geographically distant colonies [22], while the individual’s foraging behavior and prey preference may explain the differences observed in the gut microbiota composition among the samples, despite pertaining to the same geographical area. Harbor seals feed on different preys depending on the resource availability across their home ranges [3], while their living conditions are not constant due to habitat differences among the colonies.

### Diet analysis

Diet has a great influence on the composition of the gut microbiota and is considered one of the main routes of microbial colonization [60]. The composition of this microbial community will depend, among other factors, on the type of prey consumed [23]. The identification of preys is fundamental to correlating the diet of the study organism with the presence of particular microbial groups [23].

In Mexico, studies on the harbor seal diet are a recent research topic [6] and mainly based on the identification of hard prey remains in fecal samples [7,8]. Previous diet analysis of the same fecal samples used in this study and collected from the harbor seals colonies of Todos Santos, San Jeronimo, Natividad, and San Roque islands had been undertaken via metabarcoding [9].

The present research characterized the harbor seal diet at Punta Banda Estuary colony using the same metabarcoding approach in order to complete the data required for microbiome and diet comparison, with 34 potential prey species identified from the fecal samples collected at this site. Teleosts were the main prey group consumed by the seals of this colony, followed by elasmobranchs (S4 Table). Among the preferred prey consumption, five teleost species (*Auxis rochei*, *Synodus lucioceps*, *Citharichthys xanthostigma* / *sordidus*, *Menticirrhus undulatus*, *Mugil cephalus*) and one elasmobranch species (*Raja binoculata*) were detected. No significant differences were found between the consumption of fish and invertebrates in the colonies, as reported in [9]. The differences observed in the diet depend on the frequency with which a species is consumed. Given that 26% of the prey identified, including elasmobranchs and some invertebrates, do not leave hard remains in the feces, they cannot be detected with traditional non-invasive methods.

Overall, diet analysis indicated that the harbor seal presents demersal foraging behavior. Sixty-five species and 39 prey families were present in the 20 fecal samples examined in the present study (S5 Table). The prey families commonly found were Paralichthyidae (100%, N = 20), Synodontidae (100%, N = 20), Sciaenidae (90%, N = 18), Ophidiidae (80%, N = 16), Pleuronectidae (60%, N = 12), Rajidae (60%, N = 12) and Octopodidae (45%, N = 9). The fish species *Synodus lucioceps* was found in all the fecal samples, with other preys such as *Citharichthys xanthostigma*, *Chilara taylori*, *Atractoscion nobilis*, and *Raja inornata* were found in >50% of the fecal samples. These values shown above are based on the frequency of occurrence of the prey in the fecal samples and do not represent the proportion of prey consumed.

**Fig 4:**
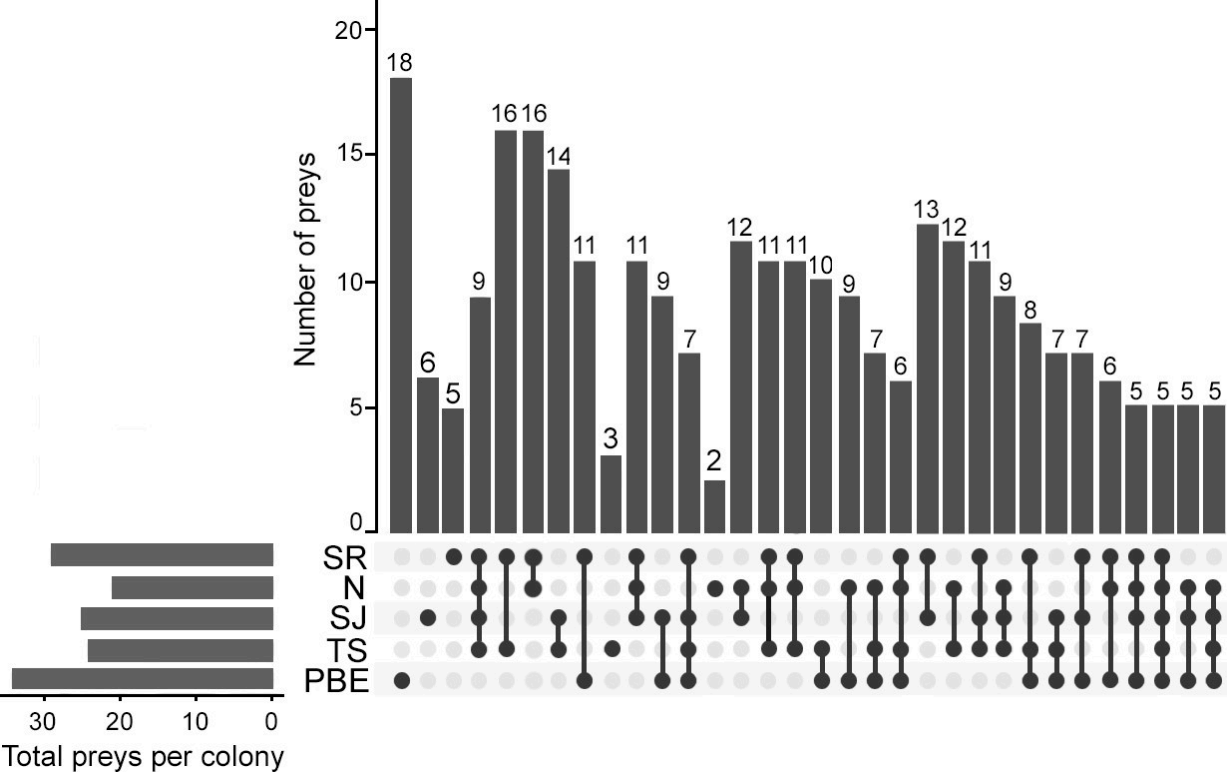
Number of unique and shared prey detected in the five harbor seal colonies. The bars on the bottom left represent the prey identified in each harbor seal colony. The upper bars represent the number unique or shared prey. The dark connected dots indicate the number of prey shared among the colonies. Not connected dots are unique prey. Data of consumed prey by harbor seal colonies from the islands Todos Santos (TS), San Jeronimo (SJ), Natividad (N) and San Roque (SR) were obtained from a previous study [9]. PBE = Punta Banda Estuary.

Compared to the other colonies, PBE presented both the highest diversity of prey consumed (Fig 4) and the highest diversity within the gut microbial community (Fig 3C and 3D). However, we did not find a correlation between prey richness and the number of microbial OTU richness (R = 0.21, p = 0.37), which is probably due to our small sample size.

### Functional prediction

We used PICRUSt to assess the metabolic potential of the gut microbiota obtained from the harbor seals. The mean value obtained via the Nearest Sequenced Taxon Index (NSTI) was 0.110, which is within the previously estimated ranges for non-human mammals [45]. This low value suggests that the predicted molecular functions of the gut microbial community are reliable [45]. We are aware that PICRUSt results are an inference and may not be a true representation of the whole metagenomic content. While ideally specific metagenome/transcriptome data should be a better option, this is not economically feasible for most laboratories, especially when examining a large number of samples. Such a project would cost thousands rather than hundreds of dollars, due to the high percentage of the host DNA as well as that of other organisms. However, a combination of constant increase in the amount of information available on various databases and the use of a reliable NSTI makes the use of PICRUSt a valuable approach, particularly in pioneering studies.

Our predictions showed that 49% of the gene families pertain to KEGG metabolism pathways while 20% were related to genetic information processing. One percent of the gene families were related to diseases (S2 Fig). The most common metabolic pathways found were those related to the metabolism of organic molecules, such as carbohydrates (22%), amino acids (20%) and lipids (6%) (S3 Fig). We found significant differences in the predicted abundance of metabolic routes among the colonies (S4 Fig), with the PBE and TS colonies presenting higher abundance in the majority of the metabolic routes when compared to the colony on SR (S4 Fig).

We identified the genes K01183 (bacterial chitinase), K01207 (β-N-acetylhexosaminidases) and K01443 (N-acetylglucosamine 6-phosphate deacetylase), which are involved in the fermentation of the polysaccharide chitin, the principal component of the invertebrate exoskeletons [61]. The genera *Bacteroides* and *Clostridium* contributed mainly to these genes and have been reported as chitinolytic bacteria in other marine mammals [62]. Bacterial chitinase may act on the chitinous crustacean exoskeletons, enabling the degradation and fermentation of individual’s diet [62,63]. It should be noted that the harbor seals consumes a lower amount invertebrates than fishes [3,9].

Within the core metabolism pathways found in all of the fecal samples, essential routes for host health and nutrition, such as those related to the metabolism of vitamins and other nutrients provided by the gut microbiota were detected [64] (S6 Table). These results suggest that microbiota play an essential role of in host’s gut metabolism and, thus, health.

We also found pathways in the core metabolism that are involved in the degradation of persistent pollutants and plastics such as atrazine [65], bisphenol [66], and chlorobenzene [67] (S6 Table). The accumulation of harmful substances has been reported in the tissues of marine mammals [68], with the degradation of these toxic compounds mainly depending on the metabolism of bacteria. The distribution of harbor seals near human settlements brings some animals into contact with humans themselves and environments influenced by terrestrial processes and human activities. The presence of pathways related to the degradation of environmental contaminants and pesticides could be due to their proximity of the colony to areas of intensive agricultural production [69,70].

**Fig 5:**
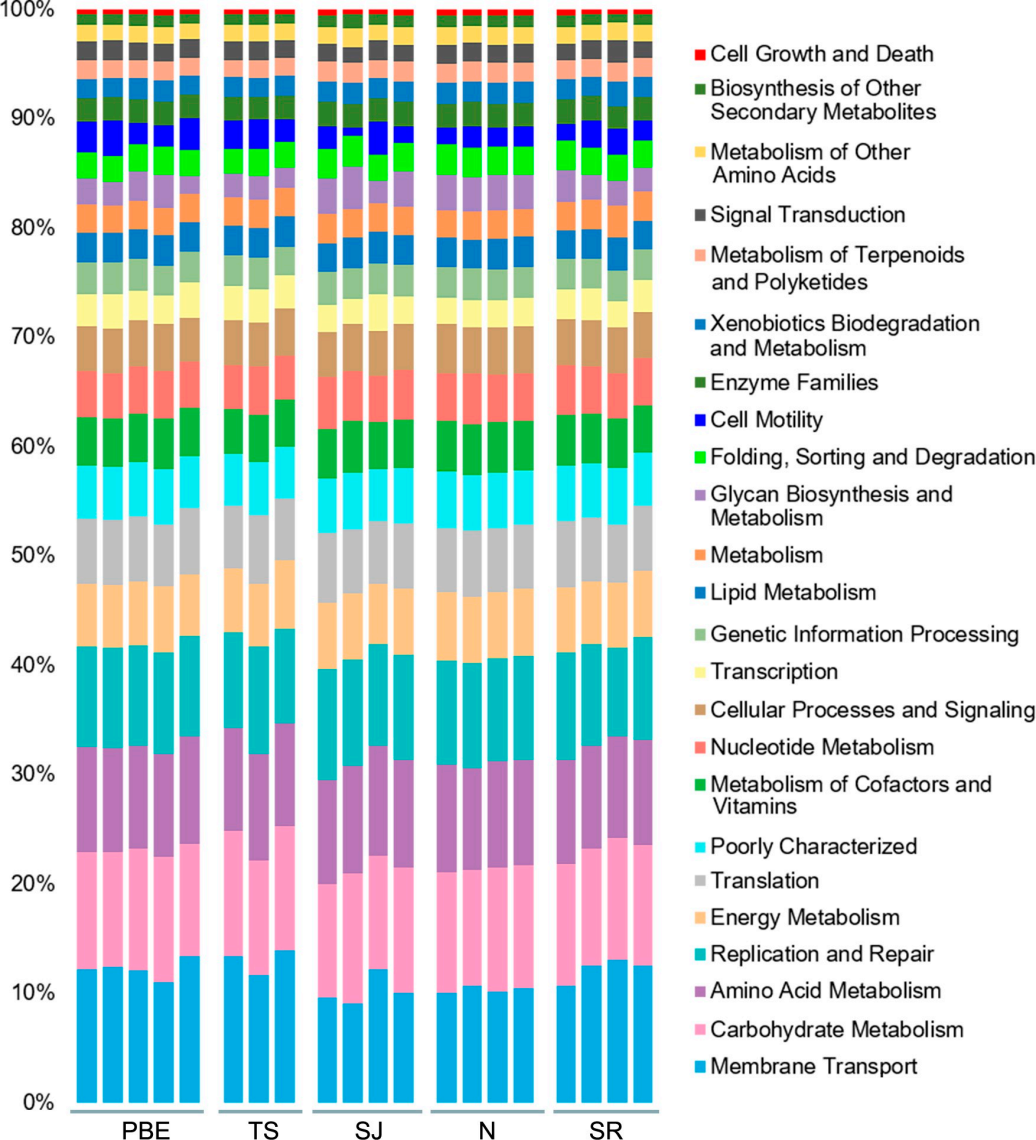
Comparisons of the principal metabolism pathways identified in the harbor seal colonies. Predicted KEEG metabolism pathways for the harbor seal microbiome using PICRUSt.

Despite the differences found for consumed prey and gut microbial community diversity among harbor seals colonies, the metabolic functions observed presented a similar pattern (Fig 5), meaning that specific conditions in the gut environment shape the microbiota metabolism in a similar way [71]. In this regard, multifunctional redundancy is an intrinsic property of the mammal gut ecosystem, thus guaranteeing a homeostatic conditions in the host [72], and was observed in the gut microbiota of the harbor seal irrespective of diet or colony location.

### Relationship between diet and microbial function and composition

**Fig 6:**
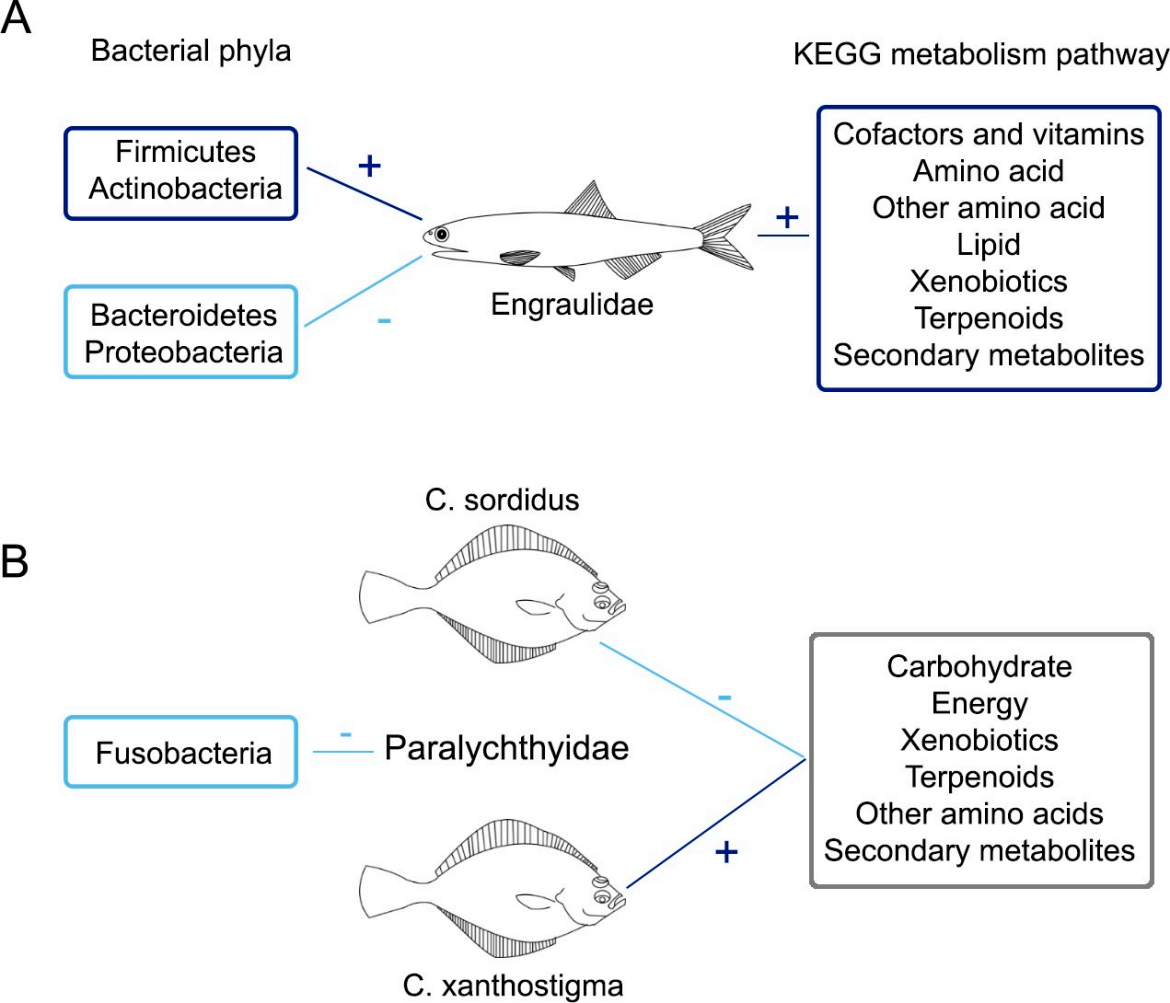
Significant correlations between prey consumed and gut microbiota function and composition. A) Relationship between the family Engraulidae and B) between members of the family Paralichthyidae. Positive (+, dark blue) and negative correlations (-, light blue) are shown.

Gut microbial composition and function change in response to diet with the structure of this micro-community rapidly altered in response to changes in macronutrient consumption [73]. We found significant correlations between the prey consumed and the bacterial composition and function of the harbor seals gut microbiome (S7 Table). The frequency of occurrence for the family Engraulidae (commonly known as the anchovy) increases the prevalence of Firmicutes (ρ = 0.585, p = 0.007) and decreases the prevalence of Bacteroidetes (ρ = -0.499, p = 0.025) (Fig 6A), and, in the present study, was represented by *Engraulis mordax* and *Anchoa compressa*. Engraulidae, which was identified in the fecal samples collected at the PBE and TS colonies, also presented high positive correlations with the main metabolic pathways, including amino acid (ρ = 0.585, p = 0.007) and lipid pathways (ρ = 0.542, p = 0.014), and a low frequency of occurrence (20%).

The anchovy is considered an ‘oily fish’ due to its higher content of polyunsaturated fatty acids, especially docosahexaenoic (DHA) and eicosapentaenoic (EPA) acids [74] than that of ‘white fishes’ (e.g. flat fish). This higher content of fatty acids may explain both the positive effect of the family Engraulidae on the relative abundance of bacteria known to be abundant in individuals with a diet rich in lipids and proteins (Firmicutes) and the family’s negative effect on bacteria associated with high-fiber diets (Bacteroidetes). Moreover, said fatty acids content may explain the high level of OTUs related to the metabolism of lipids and amino acids involving in the digestion of the prey. Our results are consistent with studies conducted on monkeys, in which energy intake was significantly related to the relative abundance of specific gut microorganisms [73,75]

The family Engraulidae was only identified in the PBE and TS fecal samples, with SIMPER analysis revealing that, in the harbor seals of these colonies, Firmicutes had the highest mean abundance (Table 1). Although Engraulidae presented a low frequency of occurrence, it is likely that consumption of this kind of prey plays an important role in shaping the function and composition in the harbor seals gut microbiota.

Contrary to our expectations, the family Paralichthyidae showed a negative correlation with the phyla Fusobacteria (ρ = -0.586, p = 0.007). Due to the demersal behavior of this fish species, we expected to find a positive influence on the relative abundance of Fusobacteria in the harbor seal microbiota due to the presence of marine sediment- associated bacteria.

At a species level, we found and inverse correlation effect for the prey species *Citharichthys xanthostigma* and *Citharichthys sordidus*. While *C*. *xanthostigma* presented high positive correlations with most of the OTUs assigned to metabolism pathways (e.g. carbohydrate, energy, and other amino acids), *C*. *sordidus* showed negative correlations (Fig 6B). *C*. *xanthostigma* demonstrated a higher frequency of occurrence (95%) than *C*. *sordidus* (30%) in the fecal samples. A preference for the consumption of *C*. *xanthostigma* may be related to it having a higher nutritional value than *C*. *sordidus*. However, given the lack of information on the nutritional values of this species, we cannot corroborate these results. Functional data should be interpreted cautiously because we do not have the nutrient consumption data required for corroborating the predicted functional pathways.

We did not find a positive relationship between the phylum Bacteroidetes and harbor seal prey at a family or species level, which is consistent with its animal protein and lipid-rich diet. It is still unclear how the prey consumed, nutrient requirements and macronutrient consumption shape the composition of the harbor seal’s gut microbial community [73].

## Conclusions

In this study, we describe the gut microbiota function and composition of wild colonies of harbor seals in Baja California. Contrary to our expectations, higher variability in the diet found in harbor seal colonies in close proximity to each other was not reflected in differences in the gut microbiota structure. We observed variation in the diversity and richness among the northern (PBE and TS) and the southern (N-TS) harbor seal colonies. The coastal harbor seal colony studied in the present research showed a higher level of gut microorganisms diversity and richness which could be explained by the acquisition of environmental bacteria due to the individuals’ proximity to the land and the higher number of prey consumed. Future research on the gut microbiota should be focused on the apparently vulnerable PBE colony in order to understand the impact of human disturbance on the composition of the gut microbiota and its probable effect on the seals’ health.

As in previous studies of the mammalian gut microbiome, our study also found correlations between diet and microbial composition. A high polyunsaturated lipid content in the prey consumed by harbor seals may be a major factor shaping the gut microbiota community and its function. Information on the nutritional values of the prey and the harbor seals’ nutrient requirements may provide insight into its prey preferences. Moreover, further metagenomic approaches would allow an understanding of the dynamic nature of the gut microbial community, and its metabolic capabilities, and role in the harbor seal’s feeding habit.

Our study represents a first insight into the factors that shape the gut microbiota of wild harbor seals, such as environmental conditions and the prey consumed. Harbor seals are non-migratory animals that forage near their colonies, due to which behavior, changes in the gut microbial composition can be attributed to changes in the location of their colonies, giving an idea of the relationship between the health status of the harbor seal and its surrounding environment. This information could not only be used to position the harbor seal as an environmental sentinel species for the Baja California coast and its islands, but may also contribute to the conservation of the subspecies.

## Supporting information

S1 Fig Harbor seal sampling locations on the western coast of Baja California. PBE = Punta Banda Estuary, TS = Todos Santos Island, SJ = San Jeronimo Island, N = Natividad Island, SR = San Roque Island.
(TIF)

S2 Fig Comparison of the bacterial phyla among the harbor seal colonies. PBE = Punta Banda Estuary, TS = Todos Santos Island, SJ = San Jeronimo Island, N = Natividad Island, SR = San Roque Island. ANOVA/Kruskal-Wallis; *p <0.1, **p<0.05.
(TIF)

S3 Fig Principal pathways identified in the harbor seal microbiome. Predicted KEGG pathways using PICRUSt for the harbor seal microbiome.
(TIF)

S4 Fig Comparison of the proportion of sequences for the metabolism pathways identified in the harbor seal microbiome. PBE = Punta Banda Estuary, TS = Todos Santos Island, SJ = San Jeronimo Island, N = Natividad Island, SR = San Roque Island. ANOVA; *p <0.1, **p<0.05.
(TIF)

S1 Table Relative abundance of bacteria in the harbor seal gut. A total of 7205 OTUs were used to construct the taxonomy table. Taxa levels include from Domain to Genus.
(XLSX)

S2 Table Comparison of the gut microbiota cores of the Pacific (the resent study) and Atlantic (Baltic study*) populations of harbor seal. (XLSX)

S3 Table SIMPER analysis comparing the gut microbiota composition of harbor seal colonies at OTU level. PBE = Punta Banda Estuary, TS = Todos Santos Island, SJ = San Jeronimo Island, N = Natividad Island, SR = San Roque Island.
(XLSX)

S4 Table Prey species identified via metagenomic analysis of the fecal samples taken from PBE harbor seals. (DOCX)

S5 Table Frequency of occurrence of the preys in the fecal samples from harbor seals. (XLSX)

S6 Table Core metabolism pathways found in all the harbor seal fecal samples. (XLSX)

S7 Table Spearman rank correlation results for diet and gut microbiota function and composition. Correlations marked in red are significant at p <0.05.
(XLSX)
