# Supplementary figures and images for: The Pacific harbor seal gut microbiota in Mexico: Its relationship with diet and functional inferences
Source: PLoS One. 2019 Aug 29;14(8):e0221770. doi: 10.1371/journal.pone.0221770 (PMC6715212; doi:10.1371/journal.pone.0221770)

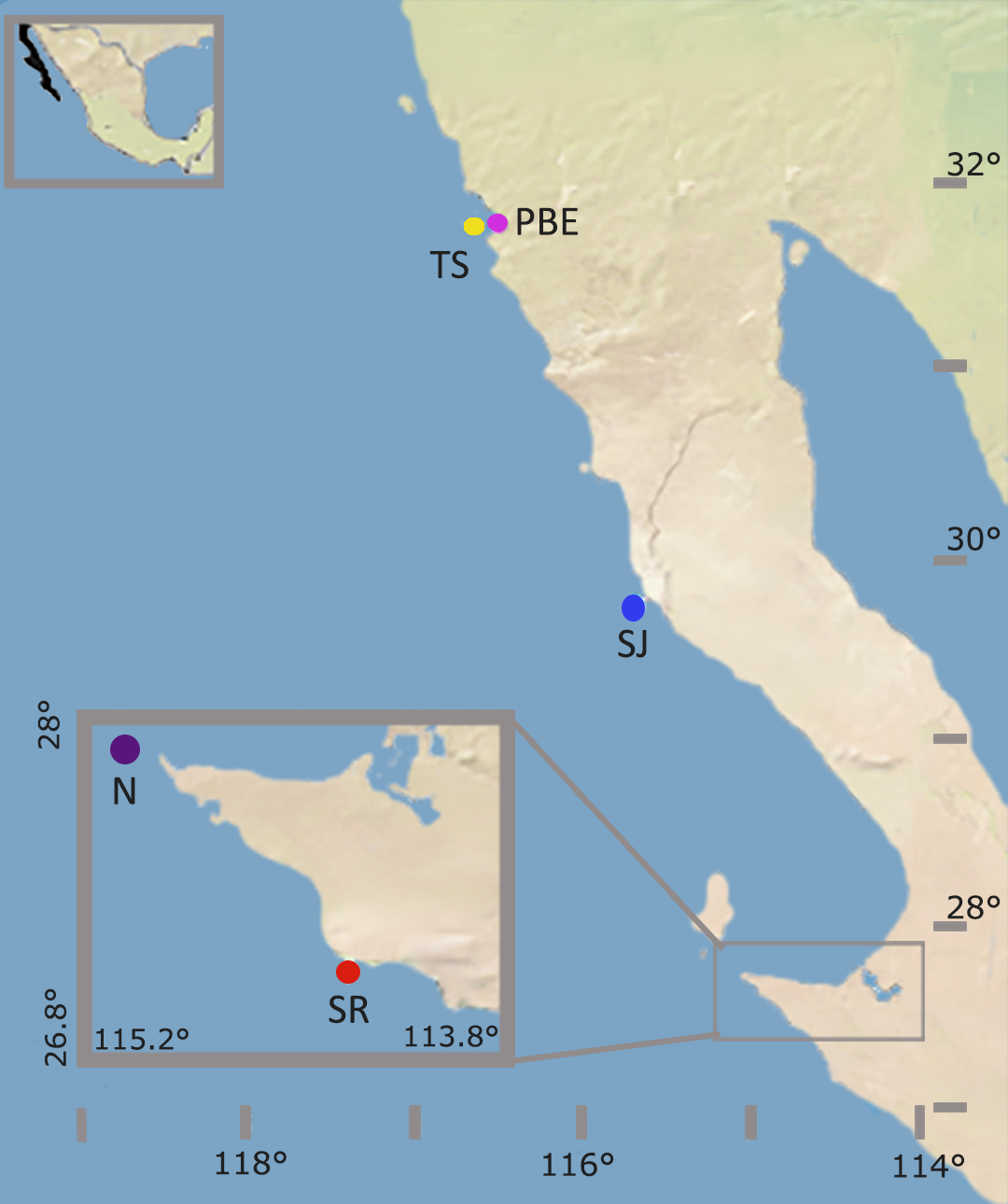

Supplement: S1 Fig — PBE = Punta Banda Estuary, TS = Todos Santos Island, SJ = San Jeronimo Island, N = Natividad Island, SR = San Roque Island. (TIF) [file pone.0221770.s001.tif]

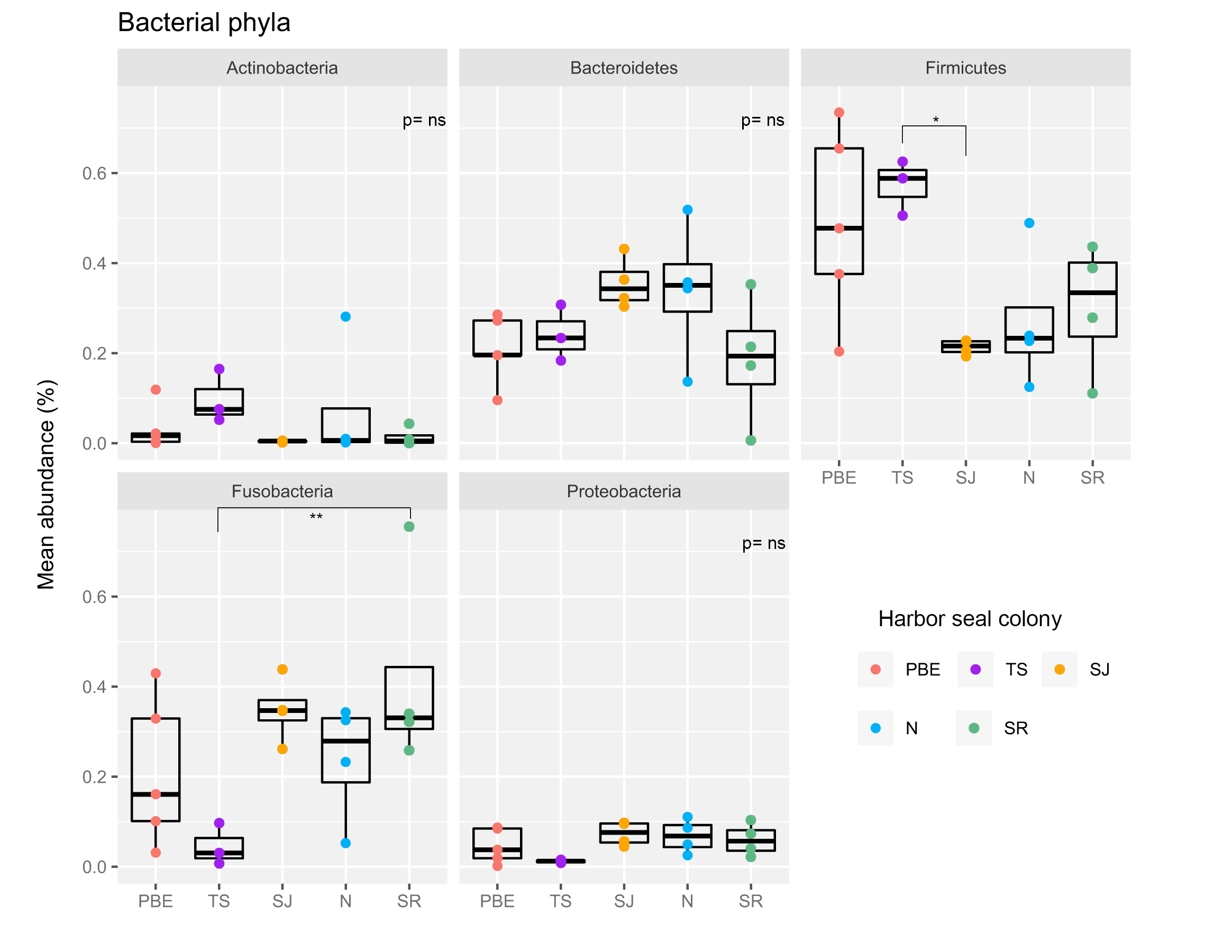

Supplement: S2 Fig — PBE = Punta Banda Estuary, TS = Todos Santos Island, SJ = San Jeronimo Island, N = Natividad Island, SR = San Roque Island. ANOVA/Kruskal-Wallis; *p <0.1, **p<0.05. (TIF) [file pone.0221770.s002.tif]

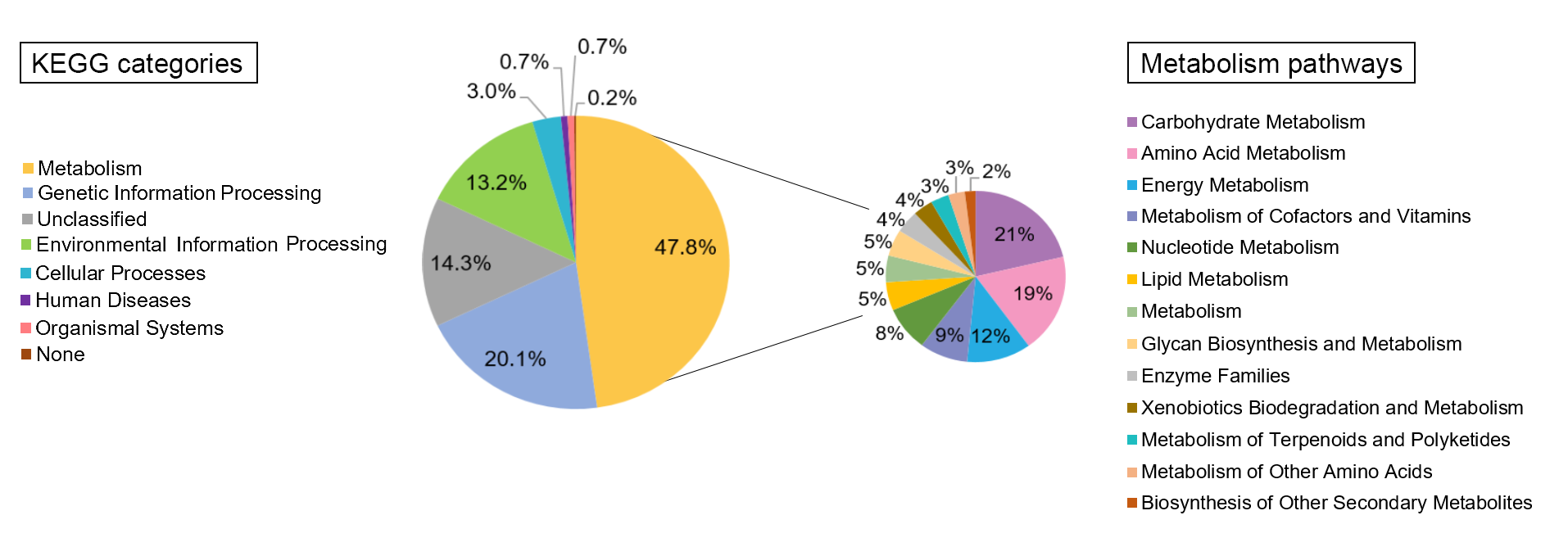

Supplement: S3 Fig — Predicted KEGG pathways using PICRUSt for the harbor seal microbiome. (TIF) [file pone.0221770.s003.tif]

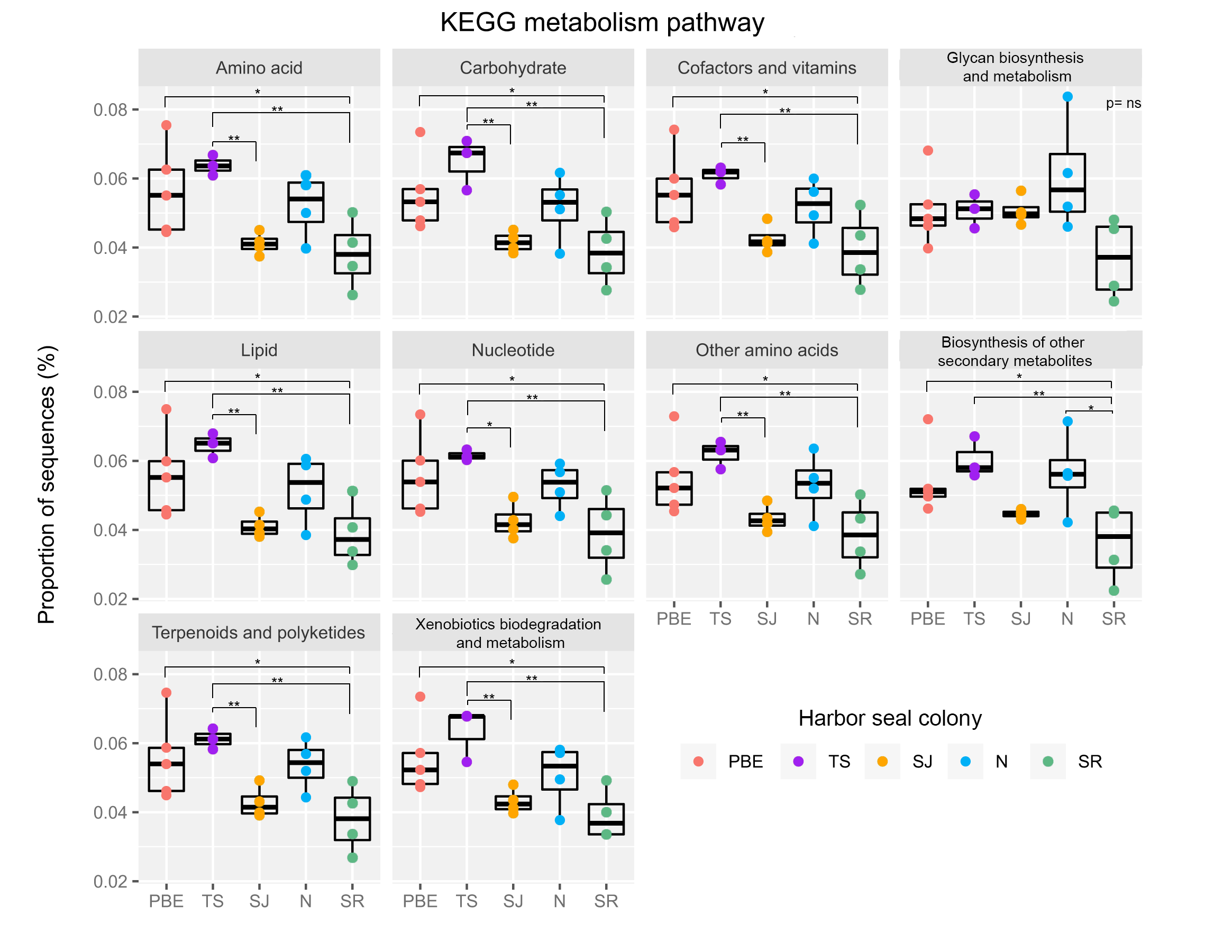

Supplement: S4 Fig — PBE = Punta Banda Estuary, TS = Todos Santos Island, SJ = San Jeronimo Island, N = Natividad Island, SR = San Roque Island. ANOVA; *p <0.1, **p<0.05. (TIF) [file pone.0221770.s004.tif]
