# Supplementary material for: The Pacific harbor seal gut microbiota in Mexico: Its relationship with diet and functional inferences
Source: PLoS One. 2019 Aug 29;14(8):e0221770. doi: 10.1371/journal.pone.0221770 (PMC6715212; doi:10.1371/journal.pone.0221770)
Supplement: S4 Table — (DOCX) [file pone.0221770.s008.docx]

**S4 Table. Prey species identified via metagenomic analysis of the fecal samples taken from PBE harbor seals.**

| **Species** | **Habitat preference** | **%FO*** |
| --- | --- | --- |
| *Auxis rochei* | Pelagic-neritic | 100% |
| *Synodus lucioceps* | Demersal | 100% |
| *Citharichthys xanthostigma/sordidus* | Demersal | 80% |
| *Menticirrhus undulatus* | Demersal | 80% |
| *Mugil cephalus* | Benthopelagic | 80% |
| *Raja binoculata* | Demersal | 80% |
| *Chilara taylori* | Demersal | 60% |
| *Hypsopsetta guttulata* | Demersal | 60% |
| *Paralichthys californicus* | Demersal | 60% |
| *Pleuronichthys verticalis* | Demersal | 60% |
| *Anchoa compressa* | Pelagic-neritic | 40% |
| *Blepharipoda occidentalis* | Benthic | 40% |
| *Citharichthys stigmaeus* | Demersal | 40% |
| *Genyonemus lineatus* | Benthopelagic | 40% |
| *Peprilus simillimus* | Demersal/benthopelagic | 40% |
| *Symphurus atricaudus* | Demersal | 40% |
| *Syngnathus leptorhynchus/exilis* | Demersal | 40% |
| *Aplysina sp.* | Demersal | 20% |
| *Engraulis mordax* | Pelagic-neritic | 20% |
| *Microstomus pacificus* | Demersal | 20% |
| *Myliobatis californica* | Demersal | 20% |
| *Neotrypaea californiensis* | Demersal | 20% |
| *Octopus rubescens* | Demersal | 20% |
| *Ophidion scrippsae* | Demersal | 20% |
| *Pachycerianthus fimbriatus* | Demersal | 20% |
| *Pleuroncodes planipes* | Demersal | 20% |
| *Pleuronichthys decurrens* | Demersal | 20% |
| *Porichthys myriaster* | Demersal | 20% |
| *Sebastes miniatus/pinniger* | Demersal | 20% |
| *Sebastes simulator* | Demersal | 20% |
| *Triakis semifasciata* | Demersal | 20% |
| *Rhinobatos productus* | Demersal | 20% |
| *Zaniolepis frenata* | Demersal | 20% |
| *Zaniolepis latipinnis* | Demersal | 20% |

* Frequency of occurrence in the fecal samples.
